# Adaptation and validation of the scale for the prevention and control of healthcare—associated infections among nursing students: an exploratory factor analysis approach

**DOI:** 10.3389/fpubh.2025.1537811

**Published:** 2025-04-25

**Authors:** Carlos Samuel Ramos-Meza, Yanet Castro-Vargas, Luis Alberto Chihuantito-Abal, Edo Gallegos Aparicio, Sdenka Caballero Aparicio, Miluska Frisancho-Camero, Gareth Del Castillo Estrada

**Affiliations:** ^1^Department of Stomatology, Andean University of Cusco, Cusco, Peru; ^2^Department of Psychology, Andean University of Cusco, Cusco, Peru; ^3^Department of Nursing, Andean University of Cusco, Cusco, Peru; ^4^Department of Human Medicine, Andean University of Cusco, Cusco, Peru

**Keywords:** healthcare-associated infections (HAIs), nursing students, exploratory factor analysis (EFA), instrument validation, prevention and control

## Abstract

This study aimed to adapt and validate the HAInnovPrev scale, a tool for assessing the prevention and control of healthcare-associated infections (HAIs) among nursing students. HAIs are a significant global health concern, particularly in healthcare education, where student training and institutional protocols must align to prevent infections effectively. The development process employed a quantitative approach, utilizing exploratory factor analysis (EFA) on data collected from 347 nursing students across two universities in Peru. This method refined the instrument, reducing the original 15 dimensions to 11 well-defined factors, encompassing key aspects such as institutional compliance, personal motivation, emotional exhaustion, and adherence to hygiene protocols. The results demonstrated that the instrument’s 11-dimensional structure was statistically valid, with measures such as the Kaiser-Meyer-Olkin (KMO) test and Bartlett’s sphericity indicating suitability for factor analysis. Internal consistency reliability, evaluated through omega coefficients, showed strong reliability for most dimensions (*ω* > 0.70). The findings suggest that the HAInnovPrev scale is a valid and reliable tool to assess critical areas of knowledge, attitudes, and practices regarding HAI prevention among nursing students. Streamlining the scale from 15 to 11 dimensions improves its practical applicability and clarity, focusing on the most relevant factors influencing students’ adherence to infection control practices. Future research should broaden the sample and include observational measures to validate these findings further.

## Introduction

Healthcare-Associated Infections (HAIs) represent a major public health concern, particularly in environments where patient care is provided over extended periods. The rise of antimicrobial resistance (AMR) has further exacerbated the challenges associated with preventing and managing HAIs, as many pathogens responsible for these infections have developed resistance to conventional treatment options (1).

In healthcare settings, the misuse and overuse of antibiotics have accelerated the emergence of multidrug-resistant organisms (MDROs), which compromise infection control efforts and increase patient morbidity and mortality (2). Studies indicate that infections caused by resistant bacteria lead to prolonged hospital stays, higher healthcare costs, and increased mortality rates, posing a significant burden on healthcare systems worldwide.

Effective prevention and control of HAIs require a comprehensive approach that integrates evidence-based infection control protocols, antimicrobial stewardship programs, and continuous education for healthcare professionals (3). Strict adherence to hand hygiene protocols, the proper use of personal protective equipment (PPE), environmental decontamination, and patient isolation measures are fundamental strategies in limiting the spread of resistant pathogens. Additionally, implementing surveillance systems to monitor antimicrobial resistance patterns and infection rates within healthcare institutions is critical for early detection and intervention.

The rise of antimicrobial resistance (AMR) has become a pressing global issue, posing a significant threat to the prevention and effective treatment of an increasing number of infections. This growing crisis presents an ongoing challenge for clinical microbiologists and infectious disease specialists (1).

According to the World Health Organization (WHO), without proactive and coordinated global action, AMR-related deaths could surpass cancer-related mortality by 2050, signaling an impending public health catastrophe (2, 3). Appropriately administering appropriate antibiotic therapy is the cornerstone of managing severe infections (4).

Empirical therapy, the initial antibiotic regimen chosen without definitive pathogen identification and susceptibility testing, plays a pivotal role in treatment strategies (5). However, it is estimated that up to 50% of all prescribed antibiotics are either unnecessary or suboptimally administered (2). This alarming statistic underscores the urgent need to refine prescribing practices and reduce the misuse of antimicrobial agents to combat the spread of resistance.

AMR is defined as the ability of various microorganisms—bacteria, viruses, parasites, and fungi—to resist the effects of antimicrobial agents designed to eliminate or inhibit them (6). According to the 2017 report by the European Commission, AMR is responsible for an estimated 700,000 deaths annually, with approximately 25,000 deaths reported each year in the European Union (7). Within the EU, approximately 3.8 million patients acquire nosocomial infections annually, leading to an estimated 33,000 deaths attributed to diseases caused by resistant bacteria (6). Between 1990 and 2021, global estimates of bacterial resistance to antibiotics revealed a decrease in seven regions and an increase in fourteen, with an excess of 10,000 deaths attributable to antimicrobial resistance (AMR) in tropical Latin America, Western Sub-Saharan Africa, North America, South Asia, and Southeast Asia. This alarming trend highlights the urgent need for enhanced global surveillance and intervention strategies to mitigate the rising burden of AMR-related mortality (8–10).

In recent years, Peru has experienced a notable increase in scientific research, particularly concerning critical priority bacteria. However, this growth is predominantly concentrated in the capital city, Lima, leading to an underrepresentation of studies from other regions. Additionally, limited funding from Peruvian institutions poses significant challenges to expanding research efforts nationwide (11).

Under normal circumstances, resistance arises naturally, but its rates are exacerbated by excessive or inappropriate antibiotic use and polypharmacy. Additional contributing factors include poor adherence to prescribed antimicrobial regimens and the prolonged use of prophylactic antimicrobials, which accelerate the development and emergence of resistant bacterial strains (6, 12, 13).

Infections caused by AMR bacteria result in significant clinical and economic consequences, including prolonged hospitalizations, treatment failures, increased healthcare costs, and higher mortality rates. Addressing the dual crises of AMR and healthcare-associated infections (HAIs) requires a comprehensive understanding of the acquisition and spread of resistance patterns. This knowledge was essential for optimizing antimicrobial prescribing practices and mitigating the escalating threat of resistance (6, 14, 15).

Healthcare-associated infections (HAIs) refer to diseases or conditions caused by infectious agents or their byproducts linked to exposure to healthcare settings, procedures, or treatments. When such exposure occurs specifically in a hospital setting, the infections are often called nosocomial infections (NIs).

The concept of healthcare-associated infections has evolved significantly beyond the confines of hospital settings. HAIs now encompass infections that patients may develop due to healthcare services in hospitals, outpatient care settings, and long-term care facilities. This broader definition contrasts with the earlier term nosocomial infections, derived from the Latin *nosocomial* (hospital), which exclusively referred to infections acquired within hospital environments (16).

HAIs represent a significant global public health challenge, ranking among the leading causes of nosocomial morbidity and mortality. They also impose a substantial economic burden on healthcare systems and patients. The consequences of HAIs include (i) extended hospital stays, (ii) increased use of antibiotics, (iii) the need for additional surgical interventions, and (iv) broader social and personal impacts.

Infections caused by antibiotic-resistant bacteria exacerbate these challenges, associated with even higher morbidity, mortality, and costs (17).

The Prevalence of Nosocomial Infection Study [EPINE, by its acronym in Spanish (*Estudio de Prevalencia de Infecciones Nosocomiales*)], conducted annually for over two decades in Spain, has provided valuable insights into the incidence of healthcare-associated infections (HAIs). According to EPINE, the prevalence of HAIs in Spain was reported at 9.87% in 1990 (18) and declined to 7.03% in 2019 (19).

This trend reflects a notable improvement in infection control practices over the years. However, despite the reduction, the persistence of HAIs underscores the ongoing challenges in eliminating these infections. The findings highlight the critical importance of sustained efforts in surveillance, prevention, and implementing evidence-based practices to reduce HAI prevalence further and enhance patient safety in healthcare settings.

This shift in understanding reflects a new paradigm in infection control that requires rethinking strategies and practices to address the complexities of healthcare delivery across diverse settings. It underscores the need for innovative approaches to collecting high-quality, standardized, and representative data (20). Such data is essential for accurately identifying infection trends, evaluating risk factors, and implementing effective prevention measures tailored to various healthcare contexts. This expanded perspective emphasizes the importance of system-wide collaboration and consistent methodologies to ensure comprehensive and impactful infection control efforts.

While some studies have examined the prevalence and resistance patterns of significant microorganisms, few have explored tools for assessing healthcare personnel’s skills in infection prevention and control. This gap in the literature highlights the need for more detailed research into using a better instrument to inform strategies to mitigate hospital care- associated infections and their clinical and public health consequences.

Various instruments are used to assess competencies related to nursing tasks; however, they do not all aim to achieve the same results as the present study, differing significantly in their components and areas of interest. Among them are the *Self-Assessment of Nursing Informatics Competencies Scale (SANICS)*, adapted into Korean (*K-SANICS*) (21), and the *Case Management Competence Scale* (22) in Asia; the *Ambulance Nurse Competence (ANC)* (23), the *Professional Nurse Self-Assessment Scale (ProffNurse SAS)* (24), the *Evidence- Based Practice Questionnaire (EBPQ-19)* (25), and the *Nursing Competence Self-Efficacy Scale (NCSES)* (26) in Europe.

In Latin America, the most geographically relevant instruments include the *Questionnaire of Competencies in Oncology* (27) and the *Evaluation of Determinant Factors Contributing to the Comprehensive Training of Social Service Interns (FDFIPSS)* (28). However, despite analyzing various knowledge, skills, and aptitudes in nursing education and professional practice, these instruments do not directly assess competencies related to the detection and management of antimicrobial resistance (AMR). Instead, there is a growing emphasis on publishing clinical studies focused on rapid detection methods (29) and prevention through the use of artificial intelligence and digital health technologies (24).

This article had two main objectives. First, it aimed to evaluate nursing students from two universities in southern Peru using an original questionnaire consisting of 15 dimensions and 93 items on the prevention and control of healthcare-associated infections (HAIs). This original instrument was designed for the ERASMUS Project on Capacity Building in Higher Education, under the project Empowering Nursing Higher Education with Innovative Healthcare-Associated Infection Prevention and Control Practices in Latin America, with reference number 101083115. Second, it examined 15 constructs that supported the responses of hundreds of nursing students, analyzing these constructs based on the students’ reactions.

## Materials and methods

The adapted instrument, HAInnovPrev Scale for Prevention and Control of Healthcare- Associated Infections Among Undergraduate Nursing Students, is a self-report questionnaire that underwent a back-translation process to convert it from English to Spanish and was validated by a panel of five experts in the field. Its adaptation and construct validation were carried out through an exploratory factor analysis (EFA), allowing for the identification of item sets, underlying dimensions of the studied construct, and the assessment of item validity using statistical methods in the Peruvian context.

The study analyzed 347 questionnaires randomly collected from students at two Peruvian universities, following the methodological recommendations of Yurrebaso-Macho et al. (30). The evaluation was conducted in the city of Cusco, the third most populous city in the Peruvian highlands and home to the Universidad Andina del Cusco, which is part of the HAInnovPrev project. Of the 347 questionnaires completed by students from the second to eighth academic cycles, with an average age of 21 years, 147 responses came from the Universidad Andina del Cusco and 200 from the Universidad Tecnológica de los Andes. Previous research by Ferrando et al. (31) suggests that a sample size exceeding 200 can minimize sampling error when performing factor analyses to establish valid evidence in measurement instruments.

The primary goal of the data analysis was to assess the psychometric properties of the instrument and gather valid evidence based on content and internal structure (32). To evaluate the internal structure of the instrument and item performance, an exploratory factor analysis (EFA) was conducted, following the recommendations of Watkins (33).

Before conducting the EFA, the Kaiser-Meyer-Olkin (KMO) measure was used to assess sampling adequacy, with a minimum acceptable value of 0.7 (34). Additionally, Bartlett’s test of sphericity was performed to confirm that both conditions for EFA were met (35). Given the ordinal nature of the data, a polychoric correlation matrix was used for the EFA (33). Parameter estimation was carried out using the Weighted Least Squares Means and Variance Adjusted (WLSMV) method, which is robust to non-normal multivariate distributions and well-suited for Likert-type scales (36). One critical methodological decision in EFA is determining the number of factors to retain. For this purpose, a parallel analysis (PA) was conducted, as recommended by Abad et al. (37).

An oblimin oblique rotation was applied to enhance interpretability, as the goal was to simplify the factor structure while allowing for correlations among factors (35). Items with factor loadings of 0.4 or higher were retained as indicators of meaningful contribution to the factors (38). The instrument’s reliability was evaluated using the internal consistency coefficient omega (*ω*), with values >0.70 considered acceptable (39, 40).

Data processing and EFA results were obtained using R software (41). The analysis was performed using the following R packages: psych (version 2.4.6) for psychometric evaluation (42), GPArotation for factor rotation (43), and lavaan for structural equation modeling (44). This robust methodological approach ensures that the adapted instrument meets rigorous psychometric standards and is suitable for use in the Peruvian context.

## Results

Several steps were undertaken to explore and refine the functionality of the items comprising the HAInnovPrev instrument. The initial step involved the selection of items from the HAInnovPrev scale. This comprehensive tool is designed to evaluate multiple dimensions of knowledge, attitudes, practices, and challenges nursing students face in preventing and controlling healthcare-associated infections (HAIs). The scale encompasses 15 key dimensions, including institutional education on HAI prevention, practical competency development, clinical practice environments, cultural attitudes toward prevention, personal attitudes, and motivational factors. The instrument assesses experiences ranging from theoretical instruction to real-world application in clinical settings using Likert-scale items. The HAInnovPrev scale is a robust measure for identifying training gaps and institutional support needs, with the ultimate goal of enhancing nursing education and improving infection control practices in healthcare environments.

After the initial review process, a preliminary scale was developed, consisting of 93 items formatted in a Likert-scale structure.

Before the methodological phase, a back-translation process was chosen for the HAInnovPrev Scale for the prevention and control of healthcare-associated infections among undergraduate nursing students. The instrument underwent back-translation, meaning it was first translated into Spanish and then back into its original language. The final process report indicated that the translation of the information contained in the instrument was very easy to understand when reverting to its original English version.

Subsequently, an expert panel review was conducted to validate content. A panel of five experts provided their opinions regarding the comprehension of the items constituting the 15 dimensions of the original instrument in Spanish. In general, they considered the instrument to be understandable and suitable for assessing the knowledge, attitudes, practices, and challenges faced by nursing students in the prevention and control of healthcare-associated infections (HAIs).

As a result, an initial EFA was conducted with all 93 items to examine the underlying factor structure. The results suggested a 12-factor model. However, some items were removed after evaluating factor loadings >0.40 (45) and identifying cross-loadings that loaded significantly on multiple factors. This refinement resulted in a revised version with 87 items. The revised instrument was subsequently sent to content reviewers for further evaluation. These experts assessed each item’s substantive interpretability, relevance, and importance. Leveraging expert reviewers is a well-established method to support scale development, particularly in specialized and underexplored areas (46). Based on this review, four items were identified as having clarity issues, leading to their removal. The instrument was further refined to include 83 items.

A second EFA was conducted on the refined set of 83 items. This analysis yielded a transparent and theoretically interpretable factor structure. The final retained version of the instrument consisted of 83 items.

This streamlined and structured approach highlights the rigorous refinement process undertaken to ensure the reliability and validity of the HAInnovPrev scale, providing a solid foundation for its application in nursing education and clinical practice research. Table 1 provides an overview of the total number of items and the decisions made throughout the process.

**Table 1:** Summary of item count and decision-making process.

Stage	Total items	Items removed	Process description
1	93	0	Initial selection of items
2	93	6	Items removed due to low factor loadings (<0.4) or cross-loadings in EFA
3	87	4	Items removed based on expert recommendations or lack of theoretical coherence
4	83	0	Final items included in the EFA analysis

The final EFA results are presented in Table 2. The descriptive measures indicate that the Kaiser-Meyer-Olkin (KMO) value, a measure of sampling adequacy, was 0.871, and Bartlett’s test of sphericity, a test of the hypothesis that the variables are uncorrelated in the population, yielded [*χ*^2^(4278) = 62,930.31, *p* < 0.000]. These findings suggest that the correlation matrix is not an identity matrix, and the variables are sufficiently interrelated to justify factor analysis. These results confirm that EFA is an appropriate modeling strategy for the dataset (47).

**Table 2:** Exploratory factor analysis results (*n* = 347).

Factor	Item	Loading	h^2^
F1 ω = 0.894	Staff members follow safety protocols.	0.796	0.651
	Hand hygiene is promoted.	0.744	0.732
	Supervisor guidance.	0.739	0.770
	Supervisor ensures PPE usage.	0.707	0.765
	Supplies for hand hygiene are available.	0.673	0.592
	Colleagues follow safety protocols.	0.649	0.521
	PPE available in key areas.	0.626	0.483
	I receive feedback if I fail to follow HAI measures.	0.608	0.611
	Alcohol-based hand sanitizers in key areas.	0.603	0.491
	Nursing team motivates adherence to HAI measures.	0.599	0.590
	Resources related to HAI.	0.535	0.591
	Visual reminders in key areas.	0.531	0.544
	“Bare below the elbows” policy.	0.516	0.362
F2 ω = 0.872	Team members think better of me.	0.851	0.525
	Team members also follow these measures.	0.778	0.478
	I will achieve better grades.	0.730	0.521
	Meeting my supervisor’s expectations.	0.722	0.594
	Driven by professional ethics.	0.717	0.671
	I want to implement prevention measures.	0.672	0.449
	Concern for patient safety.	0.596	0.608
	Aim to provide quality care.	0.557	0.501
	Concern for my own safety.	0.542	0.451
	My primary motivation is patient health.	0.527	0.586
	I understand the implications.	0.509	0.532
F3 ω = 0.843	Nurses during clinical practice.	0.793	0.625
	Simulation.	0.792	0.619
	My classmates.	0.765	0.561
	General courses.	0.753	0.553
	Supervisors during clinical practice.	0.680	0.614
	Specialized literature.	0.659	0.575
	Other professionals during clinical practice.	0.627	0.553
	HAI-specific course.	0.593	0.408
F4 ω = 0.806	Protective clothing.	0.774	0.404
	Environmental surface decontamination.	0.741	0.492
	Clinical waste management.	0.674	0.471
	Medical device decontamination.	0.668	0.467
	Use of personal protective equipment.	0.651	0.332
	Preparation and administration of intravenous medications.	0.598	0.532
	Exposure to microbial agents.	0.594	0.629
	Additional precautions.	0.565	0.523
	Hand hygiene.	0.482	0.526
	Respiratory tract infections.	0.452	0.481
	Individual risk assessment.	0.447	0.536
F5 ω = 0.841	Workshops or classes.	0.757	0.566
	Visual resources and technologies.	0.695	0.541
	Professors discuss HAI measures.	0.633	0.574
	Laboratory classes.	0.631	0.366
	Theoretical classes.	0.570	0.492
	Mandatory courses.	0.542	0.352
	Simulation.	0.529	0.541
	Adequate technological resources.	0.526	0.352
	Student-teacher relationships.	0.517	0.492
	Professors’ feedback.	0.458	0.574
F6 ω = 0.848	Highly interested in continuing a nursing career.	0.898	0.798
	Nursing fulfills my expectations.	0.863	0.718
	Satisfied with my career choice.	0.857	0.776
	Clinical practices keep me motivated.	0.792	0.663
F7 ω = 0.845	Emotional fatigue.	0.856	0.763
	Mental fatigue.	0.855	0.749
	Physical fatigue.	0.824	0.742
	Difficult moments in personal life.	0.739	0.484
	Feeling tired during clinical practice.	0.593	0.507
	Academic overload.	0.583	0.374
F8 ω = 0.828	Did not wash or disinfect hands.	0.889	0.629
	Did not use masks.	0.812	0.616
	Did not wear gloves.	0.775	0.561
	Attended a patient without changing PPE.	0.731	0.561
	Wore accessories below the elbows.	0.565	0.497
	Forgot to follow safety protocols.	0.521	0.453
F9 ω = 0.789	Caring for patients with multiple conditions.	0.815	0.661
	Managing multiple clinical tasks.	0.713	0.538
	Numerous requests from patients and relatives.	0.693	0.519
	Emotionally demanding cases.	0.641	0.483
	Conversations with peers.	0.611	0.402
	Overcrowded patient spaces.	0.532	0.424
F10 ω = 0.745	Did not wear gloves.	0.838	0.608
	Did not wash or disinfect hands.	0.822	0.653
	All HAI protocols must be followed.	0.774	0.561
F11 ω = 0.751	Some HAI protocols are unnecessary.	0.791	0.531
	I express reservations about protocols.	0.701	0.542
	Excessive use of disposable materials is unnecessary.	0.657	0.459
	PPE is uncomfortable.	0.604	0.459
	My supervisor constantly monitors my HAI measures.	0.601	0.481

Factor loadings > 0.40 are reported. h^2^ = communalities.

Factor loadings >0.40 were considered meaningful (45). The final EFA identified a structure comprising 11 factors, which explain 58% of the total variance. The communalities (h^2^), which indicate how much variance of each item is captured by the factor model, were moderate, ranging from 0.3 to 0.7. These values suggest that the factors are capturing a moderate amount of variance in the items, supporting the validity of the factor structure (34).

Regarding internal consistency, the omega coefficients (*ω*) for the 11 dimensions exceeded the suggested threshold of ≥0.70 (48). These results collectively suggest that the retained items are not only relevant but also aligned with the thresholds recommended in the literature, a fact that should instill confidence in the relevance of our research.

Consequently, the 11-factor measurement model, a robust and reliable tool, demonstrated adequate fit for the studied sample, indicating that the analyzed items reliably measured their respective dimensions. This reassures us about the reliability of our instrument. Based on the underlying factors identified and a review of the relevant literature, appropriate labels were assigned to each factor.

This robust factor structure and internal consistency provide strong evidence for the validity and reliability of the HAInnovPrev instrument in assessing multiple dimensions of knowledge, attitudes, and practices related to HAI prevention and control among nursing students.

After performing the exploratory factor analysis (EFA), the initial 15 dimensions of the HAInnovPrev instrument were refined into 11 distinct dimensions. This reduction was achieved by evaluating the statistical significance, conceptual coherence, and empirical relevance of each item. Items with factor loadings below 0.40, cross-loadings across multiple factors, or unclear theoretical relevance were removed. This streamlining process ensures that the final dimensions provide a more precise and robust framework for measuring key aspects of healthcare-associated infections (HAI) prevention and control among nursing students.

Table 3 summarizes the final 11 dimensions and describes their scope and focus. These dimensions reflect critical factors such as institutional adherence to protocols, personal motivation, and the challenges faced in clinical practice, highlighting the instrument’s practical relevance and theoretical depth.

**Table 3:** Summary of the HAInnovPrev instrument dimensions for the prevention and control of healthcare- associated infections.

Number	Dimension	Description
1	Institutional compliance with HAI protocols	Assesses adherence of healthcare institutions to HAI prevention protocols, including staff behaviors, supervisor oversight, and availability of resources like PPE and hand hygiene supplies.
2	Personal motivation and ethics	Focuses on students’ intrinsic and extrinsic motivations to follow HAI prevention measures, driven by ethical considerations, concern for patient and personal safety, and professional expectations.
3	Educational influences	Captures the impact of educational resources such as simulation-based learning, interactions with peers and supervisors, and specialized HAI training on students’ knowledge and practices.
4	Protective measures and hygiene practices	Measures students’ adherence to clinical hygiene protocols, such as the use of PPE, decontamination of surfaces, waste management, and infection risk assessment.
5	Academic resources and teaching methods	Examines the role of academic resources, including workshops, classroom teaching, and technological tools, in preparing students for effective HAI prevention.
6	Career satisfaction and commitment	Reflects students’ satisfaction with their nursing career choice and their commitment to pursuing nursing as a long-term profession.
7	Emotional and physical exhaustion	Assesses the impact of clinical practice and academic workload on students’ emotional, mental, and physical wellbeing.
8	Non-compliance behaviors	Identifies instances where students failed to follow HAI protocols, such as skipping hand hygiene, improper PPE use, or neglecting safety measures.
9	Clinical practice challenges	Highlights the difficulties faced by students during clinical practice, including multitasking, managing complex cases, and dealing with overcrowded environments.
10	Adherence to key protocols	Focuses on students’ commitment to critical HAI protocols, such as hand hygiene and PPE usage, emphasizing the importance of consistent adherence.
11	Skepticism toward protocols	Captures students’ perceptions of the practicality and necessity of certain HAI protocols, addressing concerns about discomfort or excessive use of materials.

The original 15 dimensions were condensed based on statistical evidence from the EFA. This process highlighted redundancies in items and revealed overlapping constructs, leading to the merging or elimination of items. The crucial role of expert reviewers was evident in this process, as they confirmed the lack of clarity in some items and the failure to meet statistical thresholds for factor loadings, leading to their removal.

This refined 11-factor structure enhances the instrument’s focus, ensuring it captures the most critical HAI prevention and control dimensions while maintaining theoretical and practical coherence.

## Discussion

The study makes a significant effort to address the issue of healthcare-associated infections (HAIs) among nursing students through the development and validation of the HAInnovPrev instrument. The mixed-methods approach—combining qualitative and quantitative methodologies—provides a well-rounded analysis of the dimensions underlying HAI prevention and control. By employing an exploratory factor analysis (EFA), the authors effectively assessed the instrument’s validity and reliability. This structured approach aligns with established psychometric standards, ensuring the instrument is well adapted for the Peruvian nursing student context.

One of the primary contributions of this study is its ability to reduce the complexity of the original 15-dimensional instrument into 11 refined dimensions. This reduction streamlines the tool while retaining its essence, ensuring the instrument remains focused and practically helpful for educational and clinical applications. The 11 dimensions captured critical aspects of institutional compliance, personal motivation, educational influences, adherence to protocols, academic resources, career commitment, emotional exhaustion, and skepticism toward protocols. The transition from 15 to 11 dimensions illustrates a rigorous and data- driven process to eliminate redundancy, enhance clarity, and improve overall construct validity. This refinement strengthens the practical utility of the HAInnovPrev scale for assessing nursing students’ knowledge, attitudes, and practices related to HAI prevention and control.

Another strong point of the study lies in the detailed statistical validation process. Methods such as the Kaiser-Meyer-Olkin (KMO) measure, Bartlett’s test of sphericity, and the WLSMV estimation technique contribute to the reliability of the findings. These statistical methods provided a comprehensive foundation for evaluating sampling adequacy and factor structure. Using polychoric correlations to accommodate the ordinal nature of Likert-scale responses was a crucial methodological consideration, aligning with the best practices in exploratory factor analysis.

The study also emphasizes the critical role of educational influences and career satisfaction among nursing students in determining their adherence to HAI prevention protocols. The authors make a significant observation that personal motivation, career commitment, and the presence of institutional support are essential in shaping compliance behaviors. By focusing on these aspects, the instrument highlights the need for institutions to develop environments that nurture technical competencies and intrinsic motivations. Including dimensions that capture emotional exhaustion also provides valuable insights into the factors that hinder adequate adherence to HAI protocols. Addressing these emotional and mental challenges among students is vital for developing more comprehensive infection control strategies in the future.

Despite the strengths, the study has several limitations that should be addressed in future research. While adequate for conducting exploratory factor analysis, the sample size may differ from nursing students across other geographical regions, thereby limiting the generalizability of the findings. Furthermore, focusing on students from only two universities in Peru may result in biases specific to those institutions. Future research should include a broader sample encompassing nursing students from diverse educational and cultural backgrounds to enhance the external validity of the instrument.

Another limitation lies in the reliance solely on self-reported data, which could introduce social desirability bias. It is recommended that future studies incorporate observational measures and longitudinal assessments to verify the validity of the self-reported behaviors. In addition, future research could explore the relationships between the identified factors and clinical outcomes to provide a more comprehensive understanding of the impact of HAI prevention training. Finally, while the instrument offers valuable insights into various dimensions of HAI prevention, there is scope for further development to include constructs related to interprofessional collaboration and patient engagement, which are also critical for effective infection control in healthcare settings.

In comparing the structure and metric properties of the HAInnovPrev Scale for prevention and control of healthcare-associated infections among undergraduate nursing students with other scales aimed at measuring knowledge and skills for the prevention and control of infections and diseases associated with healthcare in nursing, similarities were found with the Professional Nurse Self-Assessment Scale (ProffNurse SAS). This instrument seeks to assess the clinical competencies of nursing personnel and was designed under the Nordic model of Advanced Practice Nursing, which emphasizes the fundamental relationship between nursing staff and patients. This instrument also employed exploratory factor analysis (EFA) to evaluate the psychometric properties of the questionnaire, in addition to principal component analysis (PCA) as an extraction method, yielding six components. Two of these components aligned with the model adopted in the present study: Professional development with practical competency development and ethical decision-making with personal attitudes.

Similar results were also identified with the Questionnaire to Evaluate the Competency in Evidence-Based Practice of Registered Nurses (EBP-COQ Prof©) (49), an instrument designed to measure competencies in evidence-based practice in nursing, following the competency framework developed by Melnyk et al. (50). The statistical procedures employed included EFA with PROMAX rotation, which revealed a four-factor model. These factors linked attitudes to personal attitudes, as assumed in the instrument of the present study, and associated skills with practical competency development and the attitude factor with personal attitudes.

Additionally, similarities were found with studies that present other instruments in nursing practice, such as the Testing on Psychiatric Nurses of a Nurse Case Management Competence Scale (22), which has a more specific evaluation scope, focusing on case management competence for psychiatric nurses in Taiwan. The description of its design considered item selection based on case management practice standards and a literature review. Subsequently, content validity was achieved using the Delphi technique, and finally, the psychometric evaluation of the scale followed a cross-sectional design. Similarities were identified in the content validation process, which involved expert peer review and the use of statistical procedures. Among the dimensions that share similarities with the scale presented in this study, competence in facilitating coordination aligns with the personal attitudes factor, and competence in direct care aligns with practical competency development.

Finally, the results were compared with those of the study Determinant Factors in the Training of Social Service Interns: Construction and Validation of an Instrument (28), which represents one of the few attempts to design such instruments in Latin America. Although it does not delve deeply into statistical procedures, it proposes a 53-item questionnaire with three dimensions, where the structure dimension is linked to the clinical practice environment factor in the present study.

## Data Availability

The original contributions presented in the study are included in the article/Supplementary material, further inquiries can be directed to the corresponding author.
